# FAIR data for prehistoric mining archaeology

**DOI:** 10.1007/s00799-020-00282-8

**Published:** 2020-01-23

**Authors:** Gerald Hiebel, Gert Goldenberg, Caroline Grutsch, Klaus Hanke, Markus Staudt

**Affiliations:** 1 Surveying and Geoinformation Unit, University of Innsbruck, Innsbruck, Austria; 2 Institute of Archaeologies, University of Innsbruck, Innsbruck, Austria

**Keywords:** FAIR data, Cultural heritage, CIDOC CRM, Semantic web standards, Prehistoric mining archaeology, Prehistoric copper production

## Abstract

This paper presents an approach how to create FAIR data for prehistoric mining archaeology, based on the CIDOC CRM ontology and semantic web standards. The interdisciplinary Research Centre HiMAT (History of mining activities in the Tyrol and adjacent areas, University of Innsbruck) investigates mining history from prehistoric to modern times with an interdisciplinary approach. One of the projects carried out at the research centre is the multinational DACH project “Prehistoric copper production in the eastern and central Alps”. For a specific geographical region of the project, the data transformation to open and re-usable data is investigated in a separate Open Research Data pilot project. The methodological approach will use the FAIR principles to make data Findable, Accessible, Interoperable and Re-usable. Every archaeological investigation in Austria has to be documented according to the requirements of the Austrian Federal Monuments Office. This documentation is deposited in the CERN-based EU supported research data repository ZENODO. For each deposited file, metadata are created through the application of the conceptual metadata schema CIDOC CRM, an ISO standard for Cultural Heritage Information, which was adopted by ARIADNE, the European Union Research Infrastructure for archaeological resources. Concepts specific to mining archaeology research are organized with the DARIAH Back Bone Thesaurus, a model for sustainable interoperable thesauri maintenance, developed in the European Union Digital Research Infrastructure for the Arts and Humanities. Metadata are created through the extraction of information from the documentation and the transformation to a knowledge graph using semantic web standards. To facilitate usage, graph data are exported to hierarchical and tabular formats representing sites and objects with their geographic locations, temporal and typological assignments and links to the research activities and documents. Metadata are deposited together with the documentation into the repository.

## Introduction

**Fig. 1:**
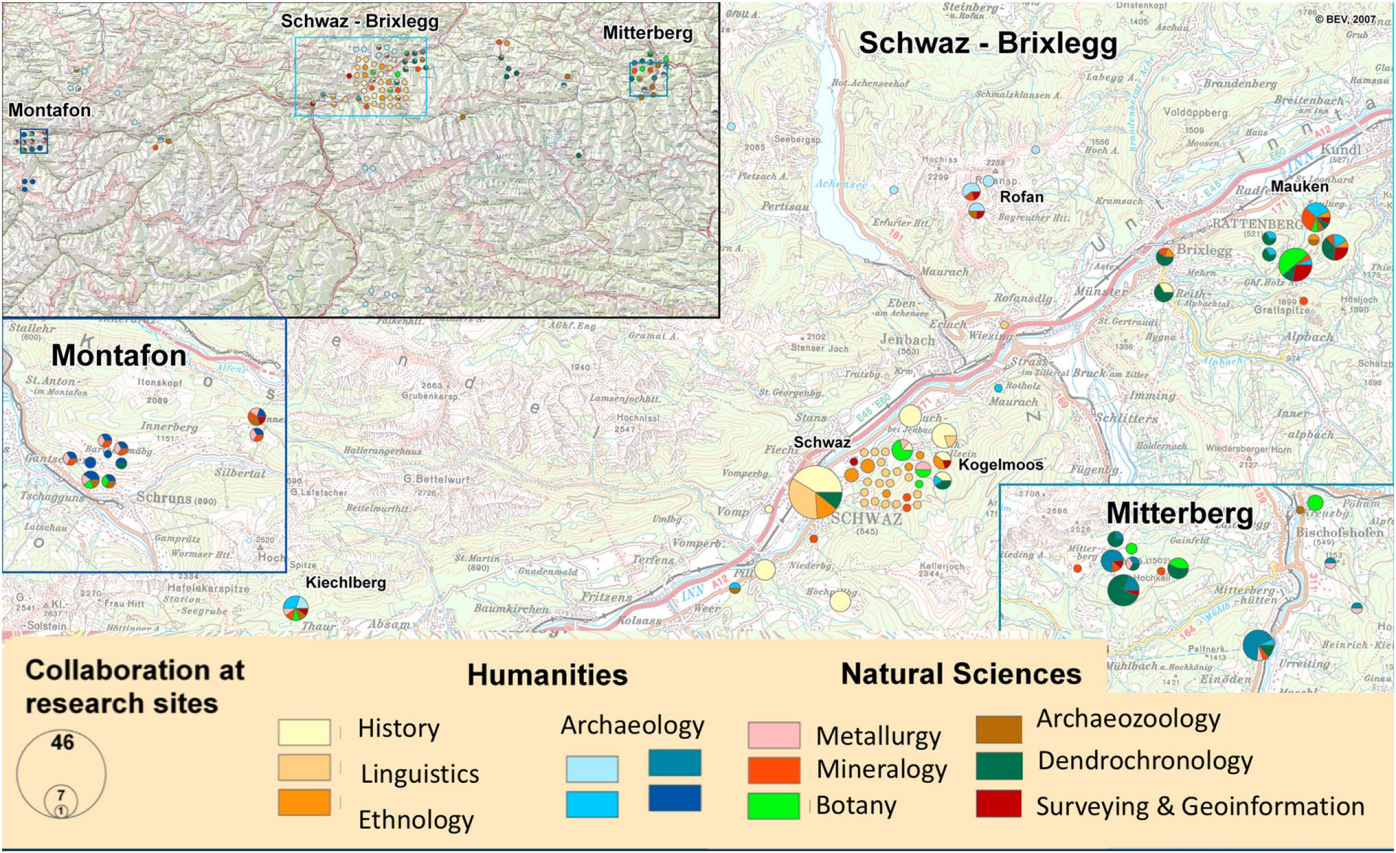
Interdisciplinary collaboration at different research sites in the frame of the special research program

Research projects produce big amounts of data which are often structured after the needs of the researching scientists and stored in institutional devices or in the cloud. In both cases, the access is restricted to specific people and re-use and evaluation of the data are limited to the people that created the data. The main reasons for this limitation are access restrictions, structure and provenance of the data which are often not sufficiently documented to re-use the data for scientific argumentation. In order to make these data or relevant parts re-useable, E-science initiatives build on the FAIR data principles [1] which state that data should be (F)indable, (A)ccessible, (I)nteroperable and (R)e-useable. As we are working with archaeological resources, the aim is to apply methodologies, guidelines and infrastructures that were created within ARIADNE, the European Union Infrastructure for archaeological resources [2]. Making archaeological datasets FAIR is one of ARIADNE’s goals [3]. In this paper, we want to present an approach of how to create FAIR data for prehistoric mining archaeology based on the CIDOC CRM ontology [4]. The implementation uses semantic web standards [5]. The approach is developed at the interdisciplinary Research Centre HiMAT (History of Mining Activities in the Tyrol and adjacent areas—impact on environment and human societies) [6] that was established at the University of Innsbruck in 2007. The main interest of HIMAT is to investigate mining history with an interdisciplinary approach. Since the start of the research centre, data related to historical mining were created by archaeological, historical, ethnological, linguistic and archaeometrical investigations (mineralogy, metallurgy, botany, archaeozoology, dendrochronology, surveying). Several projects have been realized within the Research Centre HiMAT, each of them producing data in different formats and with backgrounds in various fields of research. One of the initial projects was a 5 years Special Research Program with ten disciplines involved. Figure 1 shows the collaboration connected to different research sites. Archaeological investigations are represented by four different hues of blue in the map as different archaeological institutions were working in different areas.

From 2015 to 2018, four institutions from Germany (D), Austria (A), and Switzerland (CH) investigated mining, technology transfer and trade connections during the Bronze Age and Early Iron Age within the before mentioned multinational DACH project “Prehistoric copper production in the eastern and central Alps—technical, social and economic dynamics in space and time”. DACH is a joint funding schema of the three national funding institutions German Research Foundation (D), Austrian Science Fund (A) and Swiss National Science Foundation (CH). The Austrian part of the project has been extended with a separate Open Research Data Pilot project.

The semantic web standard for the implementation of the knowledge graph is RDF (Resource Description Framework), a data format that is able to relate logical statements within a network [7]. RDF is the foundation of the Linked Open Data (LOD) Cloud, where datasets are linked to each other on a global level. Using this format and technology means that the information within the semantic database can be linked to LOD resources, like Wikipedia or Geonames. To organize vocabularies in the documentation, SKOS (Simple Knowledge Organization System) [8] is used. This is another semantic web standard for sharing and linking knowledge organization systems, such as thesauri, taxonomies, classification schemes and subject heading systems.

To facilitate usage of the graph data, it is exported to hierarchical and tabular formats representing sites with their geographic locations, temporal and typological assignments and links to the research activities and documents. One of the products of the Open Research Data Pilot is an inventory of prehistoric mining sites that documents features, stratigraphic units and finds relating them to their investigations and the conclusions drawn. As the research data creation is based on the documentation produced for the Austrian Federal Monuments Office (mandatory for every archaeological investigation in Austria), the methodology is also applicable for other archaeological investigations in Austria. All documentation including the archaeometric analysis is deposited in a repository, after file formats have been checked for their long-term archiving suitability. The repository assigns persistent Digital Object Identifiers (DOIs) [9] and thus makes the research data citable. Metadata are created using semantic web and geoinformation technologies. The metadata are then imported into a repository, into portals and openly accessible triple stores to increase dissemination and usability. The benefit for the international research community is the possibility to analyse sites of prehistoric mining and metallurgical activities in a harmonized and reproducible format. Thus, research questions about prehistoric mining both on Alpine and European scale can be answered. For this project, Creative Commons Attribution 4.0 International (CC BY 4.0) licence is used to maximize the re-use of the data.

## Research data

**Fig. 2:**
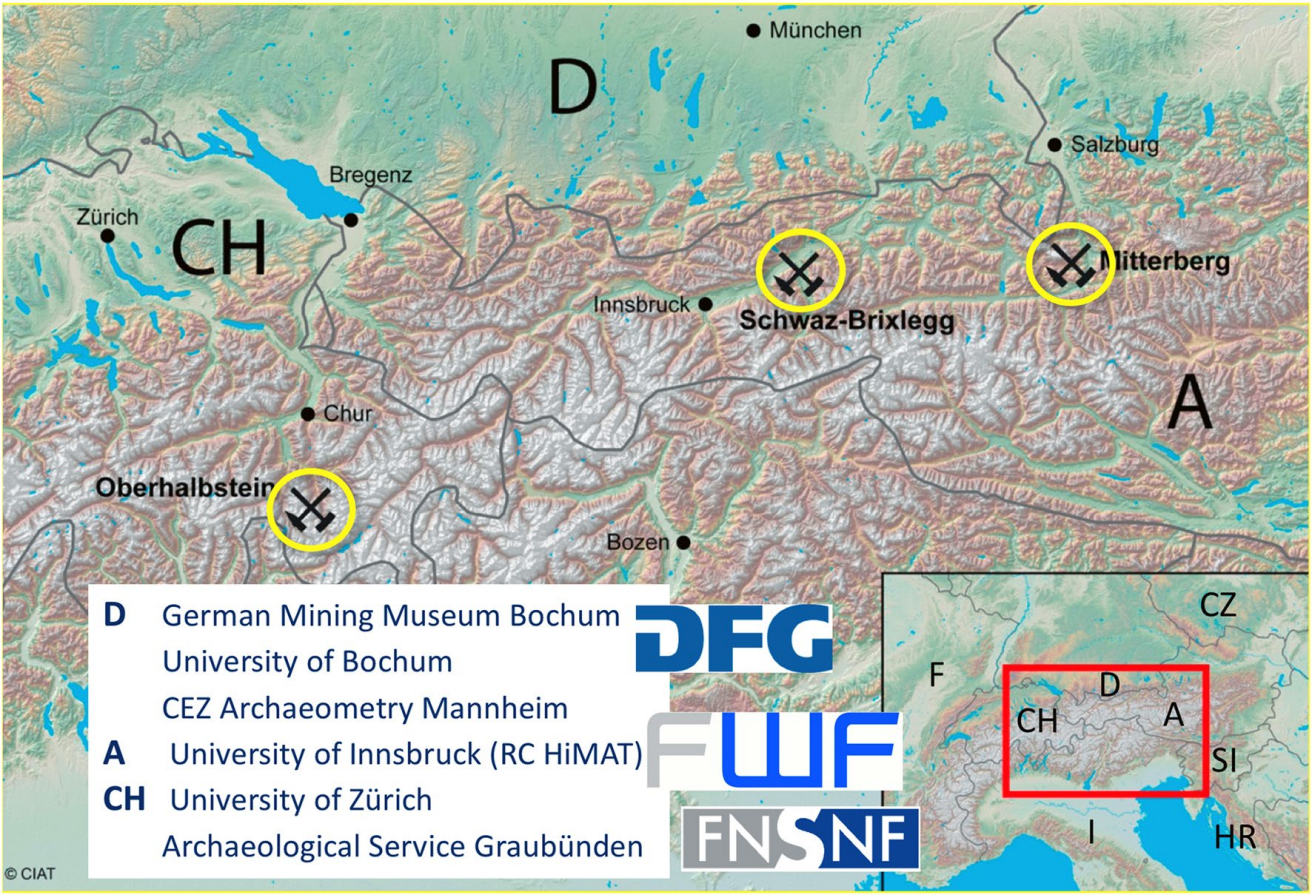
Research areas and funding institutions involved in the multinational DACH project “Prehistoric copper production in the eastern and central Alps”

The scientific data used within the Open Research Data (ORD) Pilot originate from the DACH project “Prehistoric copper production in the eastern and central Alps”. The project had the goal to reconstruct the development and influence of three mining districts of supra-regional significance, their economic dynamics and the manifold interrelations within the network of alpine metal producers. The three mining areas of Mitterberg, Schwaz-Brixlegg and Oberhalbstein together with the research and funding institutions of the project are illustrated in Fig. 2. The Mitterberg area is investigated by the German Mining Museum Bochum. It was one of the biggest Alpine copper producers, and the famous Nebra Sky Disk [10] was fabricated with its copper.

**Fig. 3:**
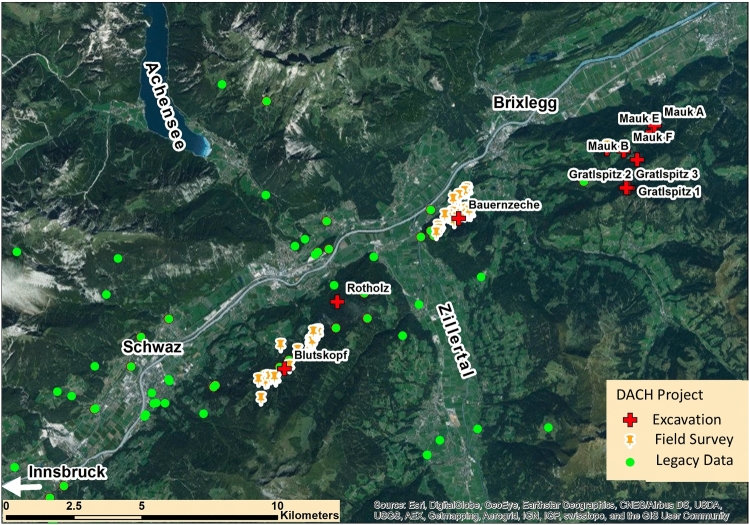
Investigated area of Schwaz-Brixlegg in the lower Inn valley as well as other excavations and surveys conducted in the course of the Research Centre HiMAT

The Austrian partner of the project investigated the mining area of Schwaz-Brixlegg. The creation of FAIR data is financed for 2 years by the Austrian Science Fund (FWF) in the course of the Open Research Data (ORD) Pilot funding schema [11]. The goal of the ORD pilot project is to digitally publish research data and to create role models for open research data. The ORD pilot project started in March 2018, and it also aims at including data from the Research Centre HiMAT. Figure 3 shows the investigated area of Schwaz-Brixlegg in the lower Inn valley as well as other excavations and surveys conducted in the course of the Research Centre HiMAT. Additionally, sites that are known from the archaeological literature are illustrated with green dots.

**Fig. 4:**
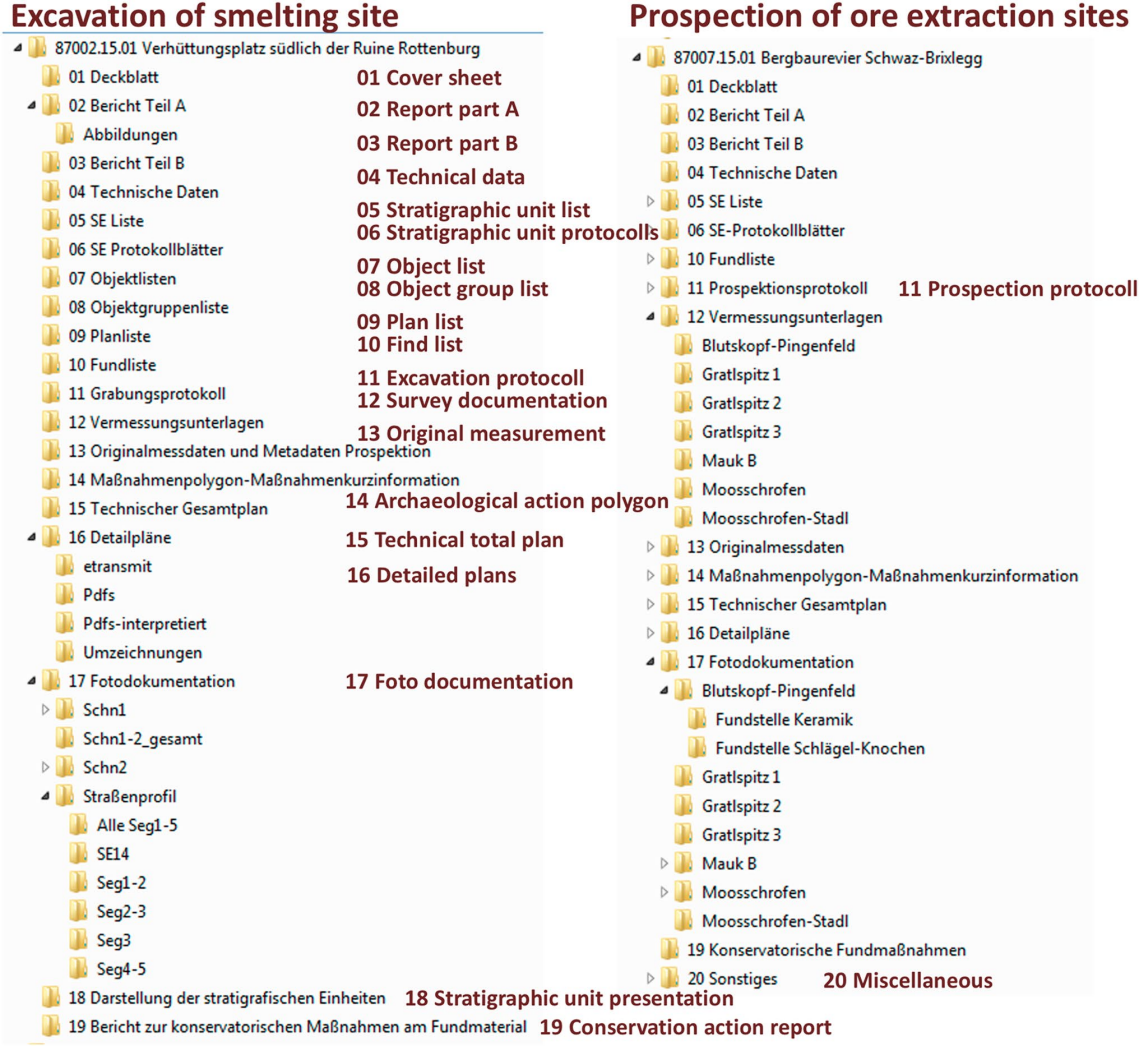
Categories and directory structure of the documentation as defined by the Federal Monuments Office. The red text translates the German folder names

For all these sites, data were created through archaeological and archaeometric investigations on physical remains of prehistoric mining and metallurgical activities. These are in particular resources created through archaeological prospections, excavations and archaeometric analysis of relevant materials and structures. Surveys try to identify sites of potential prehistoric mining and metallurgical activities. Depending on the sites, observations, surveys or even exploratory cuts are performed. Excavations and surveys are documented according to the guidelines for archaeological investigations of the Federal Monuments Office (Bundesdenkmalamt) [12]. They define in detail which reports, lists, photos and plans have to be created for prospections, excavations, stratigraphic units, finds, archaeological objects and groups. Figure 4 shows the categories and directory structure of the documentation as defined by the Federal Monuments Office. On the left side, the documentation for the excavation activity of the smelting site “87002.15.01 Verhüttungsplatz südlich der Ruine Rottenburg” is illustrated. The site was documented in 336 files with a total size of 5.34 GB. The survey activities on mining sites (underground mines and pits) in the fahlore district Schwaz-Brixlegg were documented in 244 files with a total size of 1.33 GB under the folder “87007.15.01 Bergbaurevier Schwaz-Brixlegg”. The documentation contains MS Word documents for reports and lists, pdf documents containing scans of handwritten protocols, Autodesk.dwg files in case of plans and.jpg images in case of photos.

**Fig. 5:**
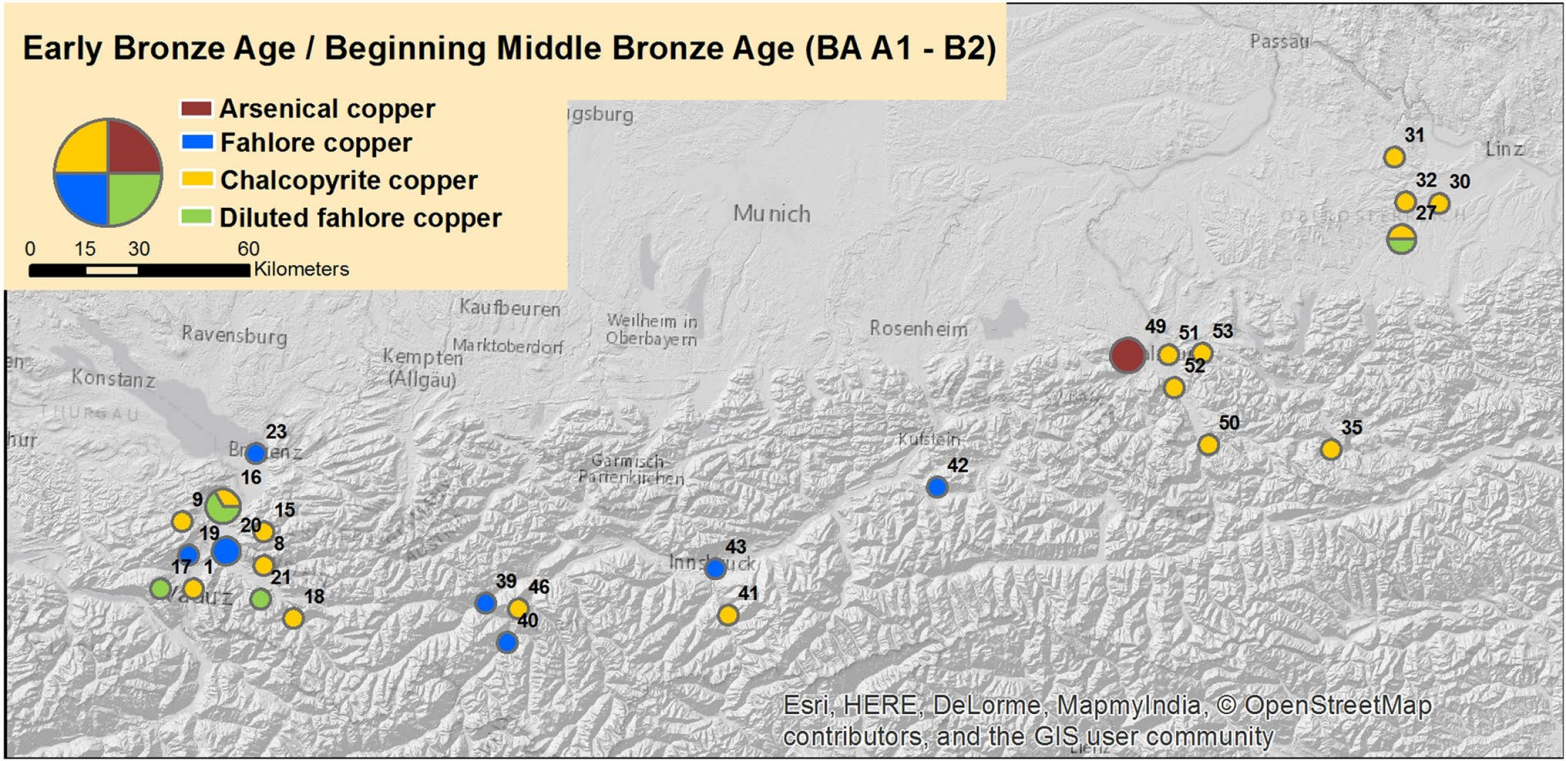
Locations of the analysed axes found in Western Austria, their copper types composition and their dating (from Early Bronze Age to the beginning of Middle Bronze Age)

Another part of the project focused on the analysis of so far 175 axes from Western and Central Austria. The goal included the increase knowledge about the type of copper that was used. The goal also included the chronological development of the use of these copper types and alloying techniques from the Early Bronze Age to the Early Iron Age mainly in Western Austria by combining archaeological with geochemical data. Figure 5 illustrates the locations of the axes, their copper type composition and their dating (Early Bronze Age—Beginning of the Middle Bronze Age). For each of the axes, X-ray fluorescence analysis has been conducted, measuring the element percentages of silver, arsenic or antimony in order to categorize the copper into arsenical, fahlore or chalcopyrite one. These analyses are documented in the form of reports and statistics, available in MS Word and PDF documents for reports and MS Excel Spreadsheets for detailed numeric and statistical information. Dendrochronological and C^14^ analyses are documented in similar ways and included in the research datasets.

In addition to the data of the “Prehistoric copper production” DACH project, relevant data from the Research Centre HiMAT were made available to provide an overview on sites of prehistoric mining activities in Austria investigated at the Research Centre during its 12 years of existence.

## Methodology to create FAIR data

The research data presented in the previous chapter are transformed to FAIR data using a specific methodology that addresses the particularly more detailed FAIR subprinciples. First, we will list them and then show which methodologies we use to realize specific subprinciples and finally create FAIR data.

### FAIR principles in detail

#### Findable

1. To be Findable any Data Object should be uniquely and persistently identifiable
2. The same Data Object should be re-findable at any point in time, and thus, Data Objects should be persistent, with emphasis on their metadata
3. A Data Object should minimally contain basic machine actionable metadata that allow it to be distinguished from other Data Objects
4. Identifiers for any concept used in Data Objects should therefore be Unique and Persistent.

#### Accessible

1. Data are Accessible to the effect that it can be always obtained by machines and humans
2. Upon appropriate authorization
3. Through a well-defined protocol
4. Thus, machines and humans alike will be able to judge the actual accessibility of each Data Object.

#### Interoperable

Data Objects can be Interoperable only if:
(Meta) data are machine actionable
(Meta) data formats utilize shared vocabularies and/or ontologies
(Meta) data within the Data Object should thus be both syntactically parsable and semantically machine accessible

#### Re-usable

For Data Objects to be Re-usable, additional criteria are:
Data Objects should be compliant with principles 1–3
(Meta) data should be sufficiently well-described and rich that it can be automatically (or with minimal human effort) linked or integrated, like-with-like, with other data sources
Published Data Objects should refer to their sources with rich enough metadata and provenance to enable proper citation.

### FAIR principles (F)indable and (A)ccessible

**Fig. 6:**
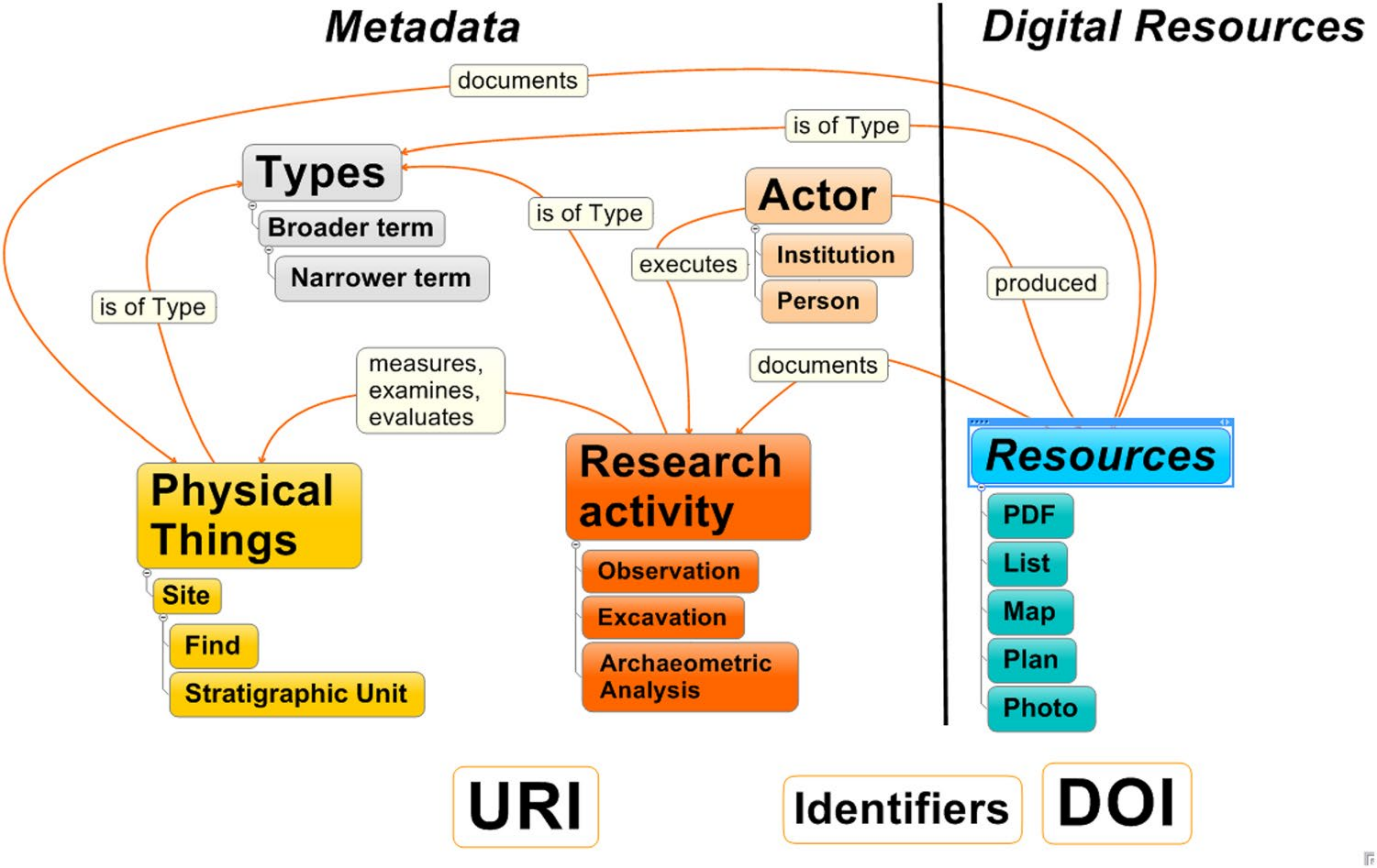
Structure of metadata (left) identified with URIs and document files (right) identified with DOIs

When describing the methodology, we will refer to the FAIR principles with their numbers stated in the previous subchapter. Research data described in chapter two are the foundation and starting point for the transformation to FAIR data. Digital resources of the Federal Monuments Office documentation are first checked for their file formats and then converted if necessary. The guidelines of Archaeology Data Service [13] and Ianus are used to generate the preferred file formats [14]. PDF/A is used for non-structured data like reports, presentations, graphics or drawings. If there are any other formats for graphical representations, they are converted to Tiff. Lists in MS Word format are converted to PDF/A and in addition to XML and CSV format. Numeric or statistic data from archaeometric analysis will be in the same formats. Autodesk.dwg files are converted to appropriate formats like SVG (Scalable Vector Graphics) and additionally will be converted to PDF/A files as well. ESRI shapefiles are created for easy use of mining site information in geoinformation applications. These digital resources are deposited in the Zenodo repository (https://zenodo.org/) located at the CERN Data Centre which has experience in long-term archiving. It is an established repository listed in the “re3data—Registry for Research Data Repositories” (https://www.re3data.org/) and is supported as a research data repository by the European Union. Zenodo assigns Digital Object Identifiers (DOIs) that are globally unique and persistent identifiers (FAIR 1), and this makes the resources accessible and citeable (e.g. reports, lists, plans from the Federal Monuments Office documentation, reports, tables or analysis from archaeometric research), according to FAIR 2. Within the Open Research Data Pilot, Uniform Resource Identifiers (URIs) for metadata entities, like archaeological sites, artefacts, actors, research activities or thesauri concepts (types), are created and linked to the above-mentioned DOIs. In order to make them globally unique and persistent, special consideration has to be taken in the URI creation process. We will try to create human readable identifiers that contain information about the object. In addition, we want to avoid duplication of the identifier and therefore assigning the same identifier as soon as the same object appears in another place. Figure 6 illustrates the relations between metadata concepts identified with URIs and digital resources identified with DOIs.

### FAIR principles (I)nteroperable and (R)e-usable

**Fig. 7:**
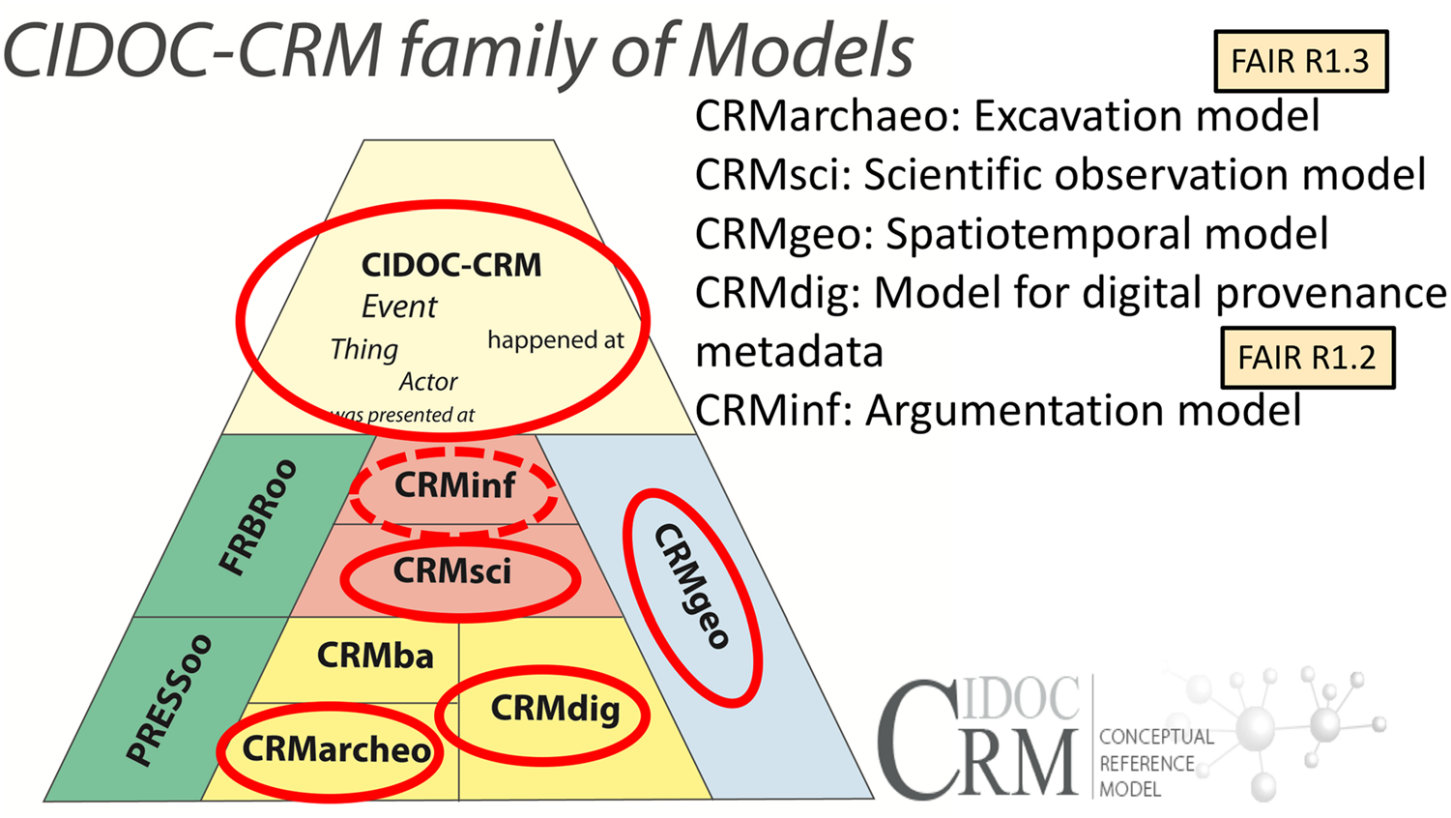
CIDOC CRM family of models

In order to fulfil the FAIR principles (I)nteroperable and (R)e-usable, metadata are encoded with the CIDOC CRM ontology which satisfies the principles to use shared vocabularies and/or ontologies (FAIR 3.2) and describe data with rich metadata (FAIR 4.2). CIDOC CRM can model the semantics of the relations between research objects, the activities and actors investigating them and the data that document the results of the investigations. Explicitly stating the research activities with their methodologies and linking them with the investigating persons and institutions will create data which is associated with detailed provenance (FAIR 4.3). Figure 7 gives an overview of the CIDOC CRM family of models with CIDOC CRM core on top and its extensions below. The red circles indicate which extensions are used for FAIR data representation to further detail the investigated objects (CRMarchaeo [15]), the activities investigating them (CRMarchaeo and CRMsci [16]), the conclusions that have been drawn (CRMinf [17]) and the data that have been created (CRMdig [18] and CRMgeo [19]).

The CRMarchaeo extension is used to model the details of archaeological excavations that are documented in the Federal Monuments Office guidelines. Archaeometric research activities like observations and measurements are modelled with the CRMsci extension. Through the CRMinf extension the observations can be separated from the interpretations. Data creation and further processing is modelled with the CRMdig extension and geographic and spatial information from surveys and excavations are modelled with the CRMgeo extension.

**Fig. 8:**
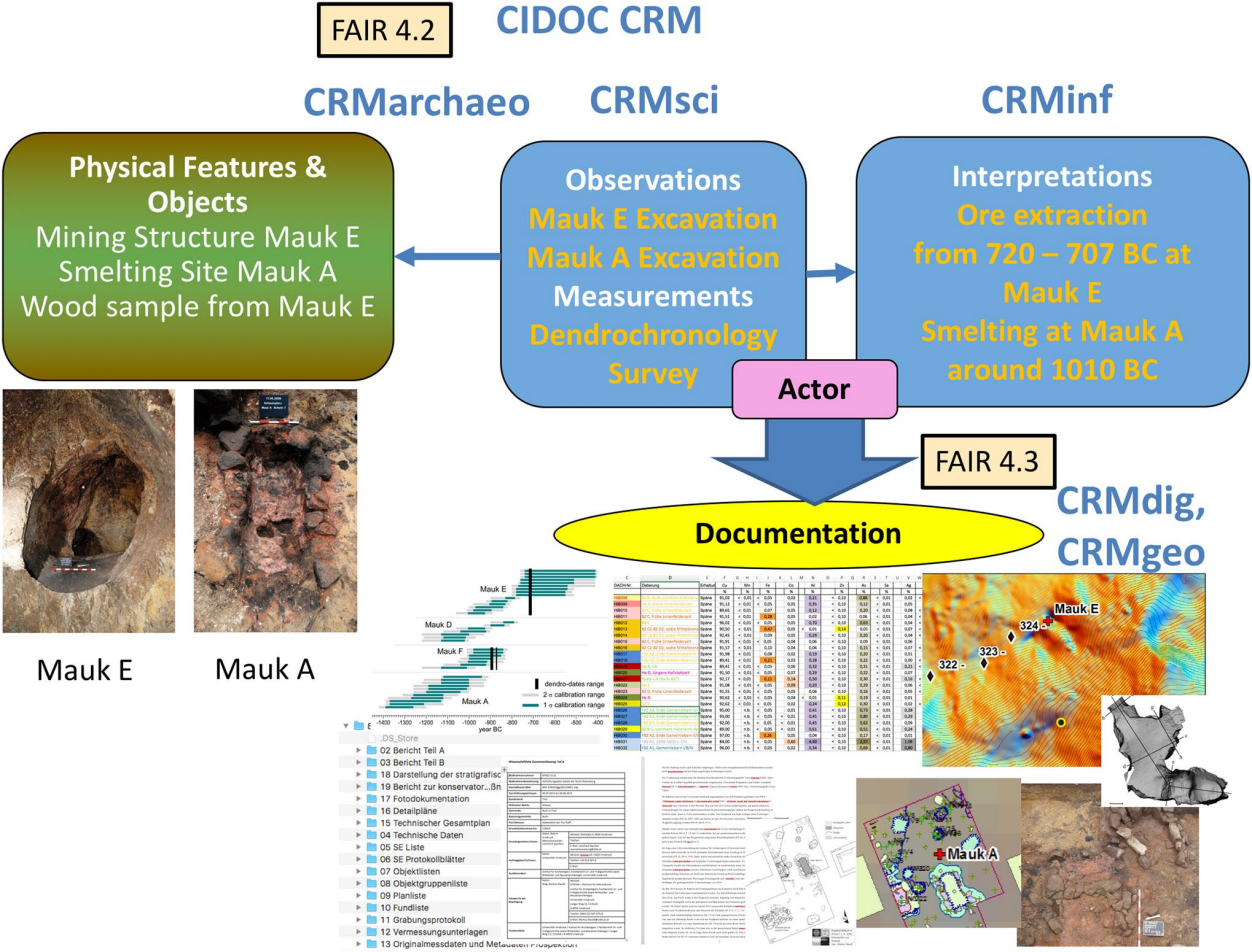
Example of FAIR data representation using CIDOC CRM and extensions

Figure 8 illustrates our modelling approach of physical features/objects, excavations, measurements, interpretations and the created documentation and data. Two archaeological features (Mauk E—ore extraction and Mauk A—smelting site) related to prehistoric copper mining and metallurgy were excavated, surveyed, and wood samples were taken. The excavated layers and finds as well as the excavation activities are modelled with CRMarchaeo. Other research activities like dendrochronological measurements, field surveys and sample taking are modelled with CRMsci. In Fig. 8, this is exemplified with Mauk E being excavated: Dendrochronological measurements have been conducted on wood samples, a 3D documentation of the mine, and the surrounding terrain was carried out. Interpretations of observations are modelled with CRMinf. Based on the dendrochronological measurements and the observations during the excavation, the archaeologists concluded that ore extraction occured at Mauk E between 720 and 707 B.C. according to the use fire setting technology. The 3D documentation as well as the created geoinformation data are related with CRMdig and CRMgeo to the research activities that created them and through them to the physical features/objects they document. Through this modelling approach, detailed provenance information is recorded.

A vocabulary is created for the terms used in the datasets, encoded in SKOS and made publicly available together with the datasets (FAIR 3.2). If terms can be identified in existing vocabularies, they are stated with “owl:same as” or SKOS relations like “skos:broader”, “skos:narrower” and “skos:related”. We will use specific facets and top concepts of the DARIAH Backbone Thesaurus [20] to structure our vocabulary.

**Fig. 9:**
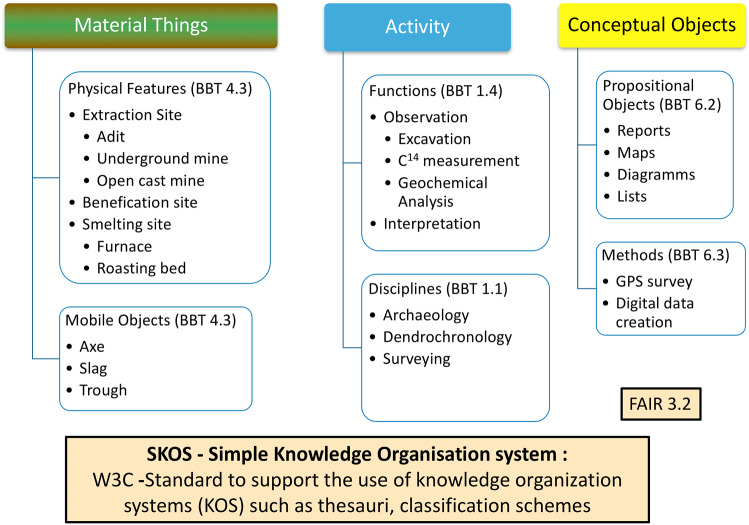
Using the DARIAH backbone Thesaurus facets and top concepts to structure the vocabulary

Figure 9 shows the facets “Material Things”, “Activity” and “Conceptual Objects” with some of the Back Bone Thesaurus (BBT) top concepts identified by their subnumbers from the BBT specification like 4.3 for Physical Features. In addition, we will use the facet “Materials” for terms like copper, fahlore, stone or wood and the facet “Types of Epochs” for Bronze Age or Iron Age and their subdivisions.

## Data structure

**Fig. 10:**
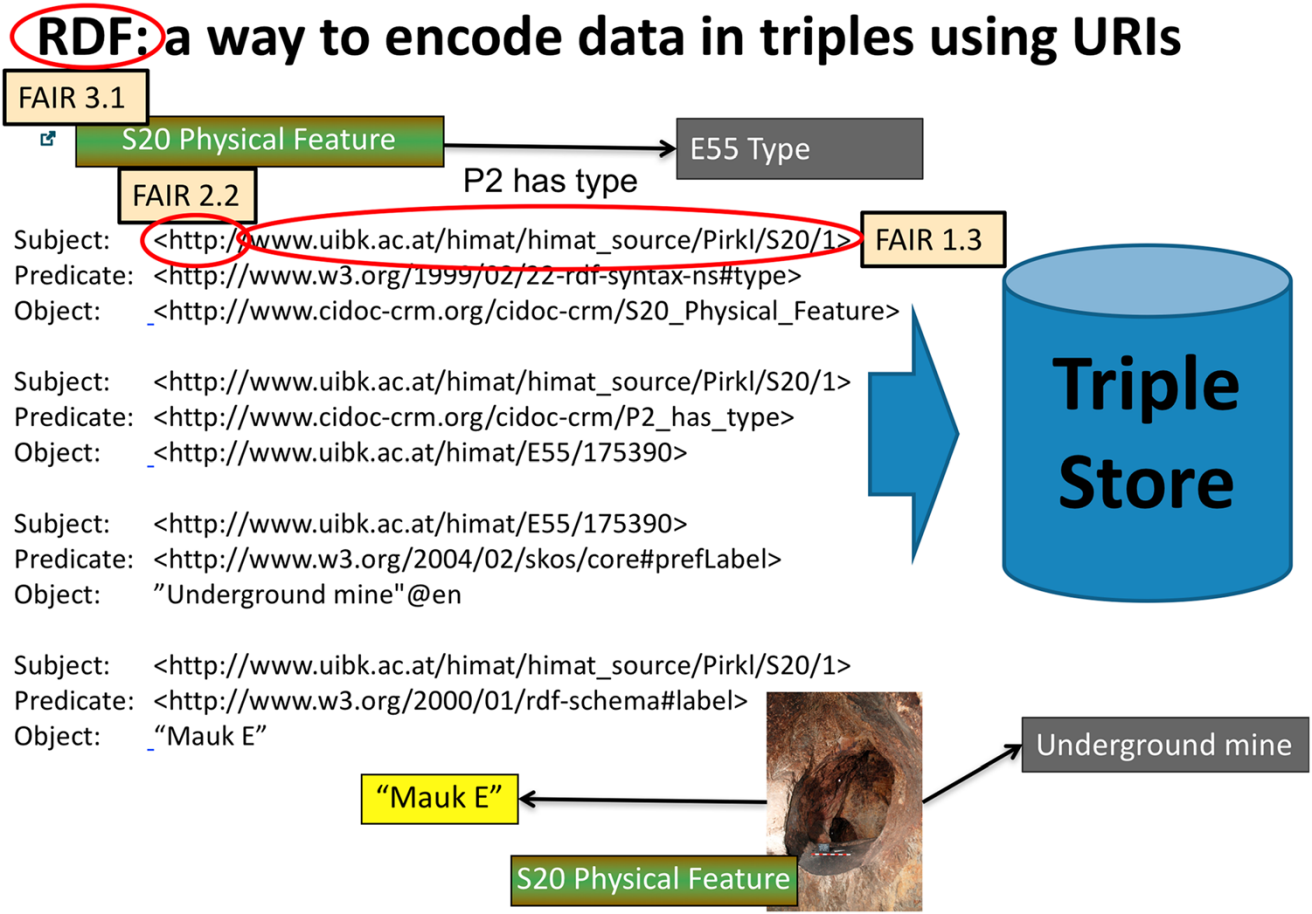
Representing metadata for “Mauk E” in RDF

To generate the metadata, the documentation created for the Federal Monuments Office is transformed to tabular data, and identifiers are created for the documented objects like sites, archaeological structures, stratigraphic units or finds. For archaeometric analysis, the creation of identifiers and tabular data is applied too, taking care that identifiers from Federal Monuments Office documentation and archaeometric analysis match. The fields of the created tables are mapped to the CIDOC CRM using tools from the semantic web community like Karma (http://www.isi.edu/integration/karma/) [21] or X3ML Toolkit [22]. Metadata are encoded in RDF using URIs and DOIs as identifiers (FAIR 1.3). Figure 10 shows metadata in RDF representing “Mauk E” as a CIDOC CRM “S20 Physical Feature” that is described as E55_Type through the SKOS concept “Underground mine”.

Document files from the Federal Monuments Office documentation and from archaeometric analysis are organized in collections according to the structure of the Federal Monuments Office. The datasets created through research activities (conducted by the same person or team) are deposited in their original form together with the created additional (tabular, GIS, PDF/A) data and the RDF files. Each collection belonging to the same research activity receives a Digital Object Identifier (DOI). If there are different files documenting one research activity, they are put together and receive one DOI as this guarantees that the executing operators will get the benefit for a citation. RDF files representing physical things, research activities, documents, actors and types (defined in a SKOS vocabulary) are attached to each collection. On the repository, the complete RDF network is deposited in an RDF dump as well as the SKOS vocabulary, both getting DOIs.

In addition to the source data, conversions and RDF, three queries are performed on the RDF network to create output in tabular, xml and JSON-LD format. The purpose of these different output formats is to minimize the threshold of using the data for different user groups. Tabular format may be used in spreadsheet applications like MS Excel, databases or geoinformation systems. Xml and JSON-LD format may be used for more sophisticated queries and tools that can handle hierarchical structured data. The first query will represent physical things like sites, finds, stratigraphic units and archaeological structures. It will contain geographic coordinate information, typologies for the physical things, relations to other physical things (part of relations), research activities and resources. Relative (through ceramics and bronze finds) and absolute datings (dendrochronology and radiocarbon dating) are related to the physical things. If available, a link to the research activity that produced them is provided. The second query will represent research activities like excavations, surveys and archaeometric analysis. It will contain actors, types for research activities and relations to other research activities (part of relations), physical things and documents. The third query will represent the vocabulary structured as a polyhierarchical thesaurus. The results of this query will always include identifiers for the objects as well as their labels, making cross-query analysis possible. The original data of the Federal Monuments Office documentation will remain unchanged, for the RDF dump, the SKOS vocabulary, and the queries’ different versions are produced if necessary and deposited on the repository with version numbers and metadata about the version.

## Conclusion

We presented an approach of how to create FAIR data for prehistoric mining archaeology derived from the Federal Monuments Office documentation which is obligatory in Austria. This means that most parts of this approach are generic and can be applied for any archaeological investigation conducted in Austria as they have to produce documentation in the same way. The methodology is based on semantic web standards to guarantee (F)indability and (A)ccessibility. To make data (I)nteroperable und (R)e-useable, data are mapped in the CIDOC CRM ontology which has been adopted by the ARIADNE EU-Infrastructure for Archaeological Resources as preferred metadata schema for Archaeology. One of the CIDOC CRM extensions used in this context is CRMarchaeo, which was build based on the official documentation requirements of different countries including the Austrian Federal Monuments Office. Therefore, the metadata standard employed here is adequate for documentation at hand and in addition a consensus on a European level. The use of CIDOC CRM as an event centric ontology enables the recording of detailed provenance which can be attached to the events of observation, measurement, interpretation and creation or modification of the documentation.

This detailed provenance is one of the keys to unfold the data re-use potential. Only if the data are trusted, it is re-used; trust is built on knowledge of the creation process of the data with the employed methodologies. The original documentation is structured in a hierarchical way with content or document types as subdivisions. Implementing an RDF network to interrelate the resources in the different subdivisions promotes potential re-use. After developing the methodology and creating RDF data for some of the resources as a test, we will now work on the dataset upload to the repository (including the necessary transformations) and creating RDF data for all the uploaded files. Research is currently in progress to apply the same methodology to the German and Swiss project partners of the “Prehistoric copper production in the eastern and central Alps” to make use of the open research data for joint analysis.

## References

[1] FORCE11: Guiding principles for findable, accessible, interoperable and re-usable data publishing version B1.0. https://www.force11.org/fairprinciples Accessed 24 Jan 2019

[2] Niccolucci, F., Richards, J.D.: ARIADNE: advanced research infrastructures for archaeological dataset networking in Europe. A new project to foster and support archaeological data sharing. In: The European Archaeologist, issue no. 39, Summer 2013 (2013)

[3] Digital Heritage Conference 2018: Are your archaeological data FAIR enough? (EU ARIADNE special session). https://digitalheritage2018.sched.com/event/HNIa/are-your-archaeological-data-fair-enough-eu-ariadne-special-session Accessed 28 Jan 2019

[4] CIDOC CRM 2018: Definition of the CIDOC conceptual reference model. http://www.cidoc-crm.org. Accessed 24 Jan 2019

[5] W3C: Semantic Web https://www.w3.org/standards/semanticweb/. Accessed 25 Jan 2019

[6] Goldenberg, G., Töchterle, U., Oeggl, K., Krenn-Leeb, A. (Hrsg.).: Forschungsprogramm HiMAT—Neues zur Bergbaugeschichte der Ostalpen. Arch. Österreich Spezial 4, Wien (2012)

[7] W3C 2014: Resource description framework (RDF). http://www.w3.org/RDF/. Accessed 25 Jan 2019

[8] W3C 2009: SKOS simple knowledge organization system reference. https://www.w3.org/TR/2009/REC-skos-reference-20090818/. Accessed 24 Jan 2019

[9] Paskin N (2006). Digital object identifiers for scientific data. Data Sci J.

[10] Pernicka, E.: Archäometallurgische Untersuchungen am und zum Hortfund von Nebra. In: H. Meller/F. Bertemes (Hrsg.), Der Griff Nach den Sternen. Wie Europas Eliten zu Macht und Reichtum kamen, pp. 719–734 (2010)

[11] Austrian Science Fund (FWF) Open Research Data (ORD): Pilot report. 10.5281/zenodo.803234. Accessed 24 Jan 2019

[12] Bundesdenkmalamt 2018. Richtlinien für ARCHÄOLOGISCHE MASSNAHMEN, https://bda.gv.at/de/publikationen/standards-leitfaeden-richtlinien/richtlinien-fuer-archaeologische-massnahmen/. Accessed 24 Jan 2019

[13] Archaeology Data Service 2019: Archaeology data service/digital antiquity guides to good practice. https://guides.archaeologydataservice.ac.uk/g2gpwiki/. Accessed 2 July 2019

[14] IANUS 2017: Archivierung bei IANUS. https://www.ianus-fdz.de/it-empfehlungen/archivierung

[15] CIDOC CRM 2018: Definition of the CRMarchaeo. An extension of CIDOC CRM to support the archaeological excavation process. http://www.cidoc-crm.org/crmarchaeo/. Accessed 28 Jan 2019

[16] Doerr, M., Kritsotaki, A., Rousakis, Y., Hiebel, G., Theodoridou, M.: CRMsci: the scientific observation model—an extension of CIDOC-CRM to support scientific observation. http://www.ics.forth.gr/isl/index_main.php?l=e&c=663. Accessed Apr 26 2018

[17] Stead, St. et al.: CRMinf: the argumentation model. An extension of CIDOC-CRM to support argumentation. http://www.cidoc-crm.org/crminf/sites/default/files/CRMinf-0.7%28forSite%29.pdf. Accessed 2 July 2019 (2015)

[18] Doerr, M., Theodoridou, M.: CRMdig: a generic digital provenance model for scientific observation. TaPP’11, 3rd USENIX workshop on the theory and practice of provenance, Heraklion, Crete, Greece, June 20–21, 2011 (2011)

[19] Hiebel G, Doerr M, Eide Ø (2017). CRMgeo: a spatiotemporal extension of CIDOC-CRM. Int. J. Digital Librar. Spec. Issue.

[20] Doerr, M., et al.: DARIAH backbone thesaurus (BBT)—definition of a model for sustainable interoperable thesauri maintenance, Produced by the thesaurus maintenance working group, VCC3, DARIAH EU, http://83.212.168.219/DariahCrete/en/bbt_intro_en. Accessed 29 Jan 2019 (2016)

[21] Knoblock, C.A., Szekely, P., Ambite, J.L., Goel, A., Gupta, S., Lerman, K., Muslea, M., Taheriyan, M., Mallick, P.: Semi-automatically mapping structured sources into the semantic web. In: Proceedings of the 9th International Conference on the Semantic Web: Research and Applications. Springer, Berlin, pp. 375–390 (2012)

[22] Marketakis Y, Minadakis N, Kondylakis H (2017). X3ML mapping framework for information integration in cultural heritage and beyond. Int. J. Digit. Libr..

